# Inclusion Complexes of Ethanamizuril with β- and Hydroxypropyl-β-Cyclodextrin in Aqueous Solution and in Solid State: A Comparison Study

**DOI:** 10.3390/molecules29102164

**Published:** 2024-05-07

**Authors:** Juan Guo, Lifang Zhang, Mi Wang, Yingchun Liu, Chenzhong Fei

**Affiliations:** 1 Shanghai Veterinary Research Institute, Chinese Academy of Agricultural Sciences, Shanghai 200241, China; hbguojuan@163.com (J.G.); wangmi@shvri.ac.cn (M.W.); liuyingchun@shvri.ac.cn (Y.L.); aries@shvri.ac.cn (C.F.); 2 Key Laboratory of Veterinary Chemical Drugs and Pharmaceutics, Ministry of Agriculture and Rural Affairs, Shanghai 200241, China

**Keywords:** Ethanamizuril, inclusion complex, β-cyclodextrin, hydroxypropyl-β-cyclodextrin, solubilization

## Abstract

Ethanamizuril (EZL) is a new anticoccidial drug developed by our Shanghai Veterinary Research Institute. Since EZL is almost insoluble in water, we conducted a study to improve the solubility of EZL by forming inclusion complexes with β-cyclodextrin (β-CD) and hydroxypropyl-β-cyclodextrin (HP-β-CD). In this study, we performed molecular docking and then systematically compared the interactions of EZL with β-CD and HP-β-CD in both aqueous solution and the solid state, aiming to elucidate the solubilization effect and mechanism of cyclodextrins (CDs). The interactions were also examined in the solid state using DSC, PXRD, and FT-IR. The interactions of EZL with CDs in an aqueous solution were investigated using PSA, UV-vis spectroscopy, MS, ^1^H NMR, and 2D ROESY. The results of phase solubility experiments revealed that both β-CD and HP-β-CD formed inclusion complexes with EZL in a 1:1 molar ratio. Among them, HP-β-CD exhibited higher *K*_f_ (stability constant) and CE (complexation efficiency) values as well as a stronger solubilization effect. Furthermore, the two cyclodextrins were found to interact with EZL in a similar manner. The results of our FT-IR and 2D ROESY experiments are in agreement with the theoretical results derived from molecular simulations. These results indicated that intermolecular hydrogen bonds existing between the C=O group on the triazine ring of EZL and the O-H group of CDs, as well as the hydrophobic interactions between the hydrogen on the benzene ring of EZL and the hydrogen of CDs, played crucial roles in the formation of EZL/CD inclusion complexes. The results of this study can lay the foundation for the future development of high-concentration drinking water delivery formulations for EZL.

## 1. Introduction

Coccidiosis in chickens is a significant global parasitic disease characterized by widespread distribution, high morbidity, and elevated mortality rates, constituting a major infectious challenge that hinders the progress of the chicken industry [1,2]. Preventing and treating this disease relies heavily on drug treatments [3,4]. Yet the widespread and prolonged use of anticoccidial drugs has resulted in the global emergence of drug resistance against all of these drugs [5]. To ensure sustainable disease control in poultry, it is imperative to develop new drugs for coccidiosis [6].

Ethanamizuril (EZL) is a new anticoccidial drug developed and synthesized by the Shanghai Veterinary Research Institute of the Chinese Academy of Agricultural Sciences in recent years [7]. It has been demonstrated that EZL is broad-spectrum, is safe, and has low toxicity, along with high anticoccidial activity, as measured by an anticoccidial index (ACI) of 185–200 [8,9].

While this drug possesses numerous advantages, its low water solubility presents a significant limitation that hinders its practical clinical application via drinking water [10]. Previous studies have reported the use of the micellar solubilization technique to improve EZL’s solubility and dissolution [11]. However, the investigation employed various surfactants, which resulted in the drug generating a substantial quantity of foam upon water mixing. Hence, it is critical to implement a better technique to increase EZL’s solubility.

Cyclodextrins (CDs) are cyclic oligosaccharides consisting of (α-1,4-)-linked α-D-glucopyranose units. They have a lipophilic central cavity and a hydrophilic outer surface [12]. They play significant roles in supramolecular chemistry due to their ability to accommodate various types of guest molecules within their CD cavity [13,14]. Upon complex formation, CD can considerably increase the solubility of weakly water-soluble substances, as well as enhance drug stability, permeability, aqueous dissolution, and bioavailability [15,16]. β-CD is a go-to choice in CD_S_ owing to its affordable price and easy availability. β-CD derivatives (such as M-β-CD and HP-β-CD) undergo chemical modification at β-CD’s hydroxyl group, offering improved water solubility and reduced toxicity as key benefits [17,18]. For example, one study found that daidzein‘s solubility increased 1474-fold after forming an inclusion complex with HP-β-CD [17]. As far as we know, there are no published scientific studies on CDs increasing the solubility of EZL. At present, only β-CD and HP-β-CD are cyclodextrin excipients included in China Veterinary Pharmacopoeia. Therefore, to secure market acceptance, we selected β-CD and HP-β-CD for our subsequent research. 

Our work aimed to systematically examine the effect of an EZL inclusion complex formed with β-CD and HP-β-CD on the solubility of EZL in both liquid and solid states. This study’s findings could lay the groundwork for the future development of high-concentration drinking water delivery formulations for EZL.

## 2. Results and Discussion

### 2.1. Phase Solubility Studies

**Figure 1:**
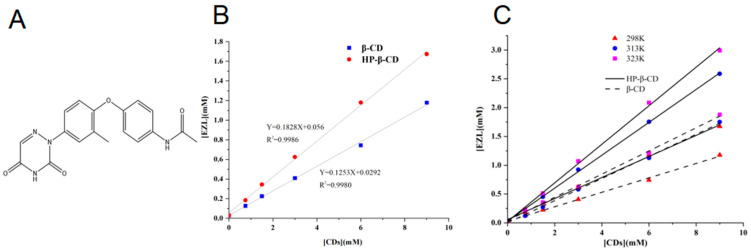
(**A**) Structure of EZL. (**B**) Phase solubility diagrams of EZL in the presence of β-CD and HP-β-CD at 298 K (25 °C). (**C**) Phase solubility diagrams of EZL in the presence of β-CD and HP-β-CD at different temperatures (298 K (25 °C), 313 K (40 °C), and 323 K (50 °C)).

Phase solubility diagrams have been widely utilized to investigate the solubilizing effect produced by different cyclodextrins on drugs [18,19]. Two unique phase solubility diagrams emerged due to variations in drug properties and cyclodextrin interactions. Solubilization occurs when a drug forms a soluble inclusion with cyclodextrin, denoted by the A-type profile. Type A is classified into A_L_ (1:1 host-guest ratio, linear growth), A_P_ (positive deviation), and A_N_ (negative deviation) types. The phase solubility curve corresponds to the B-type profile when drug solubility is limited with increasing cyclodextrin content [20]. Figure 1A,B show the structural formula of EZL and its phase solubility behavior in aqueous solutions of β-CD and HP-β-CD at 298 K (25 °C). The results showed that the apparent solubility of EZL linearly increased with the contents of β-CD and HP-β-CD, with slopes of 0.1253 and 0.1828, respectively. The intrinsic solubility (S_0_) of EZL is 0.03 mM. The results showed that the phase solubility diagrams of EZL with both β-CD and HP-β-CD were of the A_L_ type by referring to the classification criteria proposed by Higuchi and Connors [20]. This also indicates that EZL is encapsulated with both cyclodextrins in a molar ratio of 1:1.

The *K*_f_ values of EZL/β-CD and EZL/HP-β-CD were 4774.97 ± 136.24 and 7436.41 ± 91.11 M^−1^, correspondingly. The CE value for β-CD and HP-β-CD were 0.14 and 0.22, respectively. This confirms that HP-β-CD is a better solubilizer than β-CD. To be specific, the solubility of EZL increased up to 14.4 and 21.9-fold in 3 mM solutions of β-CD and HP-β-CD, respectively. In summary, the complexation of EZL with CDs dramatically improved the water solubility of the drug. In addition, we further investigated the effect of temperature on EZL complexation. The complexation efficiency increased with increasing temperature as shown by phase dissolution studies at different temperatures (Figure 1C). The study currently focuses on cyclodextrin solubilization. Future product development should account for pH and co-solvent interactions. 

### 2.2. Molecular Modeling Studies

Computer simulations offer beneficial understanding concerning guest–host interactions, enabling the theoretical validation of experimental results [21,22]. The main objective of the molecular modeling experiments in this study was to obtain an accurate representation of the complex’s structure. 

**Figure 2:**
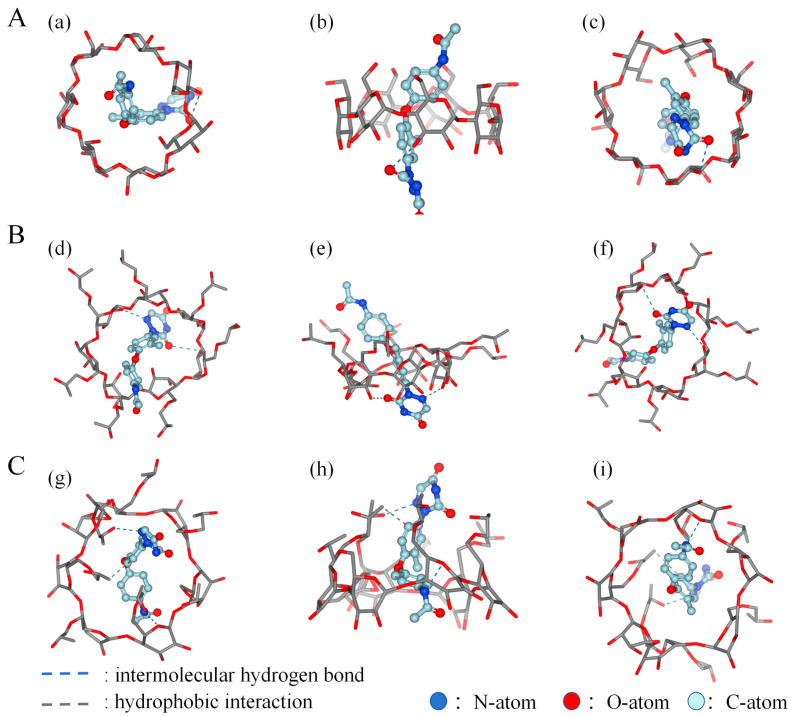
Modes of the inclusion complexes of EZL with β-CD and HP-β-CD with different orientations derived from docking simulations: (**a**) top, (**b**) side, and (**c**) bottom views of the EZL/β-CD complex (**A**); (**d**) top, (**e**) side, and (**f**) bottom views of the EZL/HP-β-CD complex (**B**); and (**g**) top, (**h**) side, and (**i**) bottom views of the EZL/HP-β-CD complex (**C**).

The phase solubility analysis established that EZL forms 1:1 complexes specifically with both HP-β-CD and β-CD. Consequently, we employed molecular docking to theoretically model the interaction between EZL and the two cyclodextrins. Figure 2 depicts the optimized geometry of the inclusion with the lowest binding energy. The optimized structure of the EZL/β-CD inclusion complex (Grid Score: −38.470371 kcal/mol) (Figure 2A) shows that an intermolecular hydrogen bond formed between the C=O group of the EZL triazine ring and the 3-OH group inside the β-CD. Similarly, the optimized structure of the EZL/HP-β-CD inclusion complex (Grid Score: −45.609547 kcal/mol) (Figure 2B) revealed two intermolecular hydrogen bonds: one formed between the C=O group of the EZL triazine ring and the 3-OH group inside the HP-β-CD, and another formed between the N-H group of the EZL triazine ring and the 2-OH group of the HP-β-CD. Furthermore, another docking result (Figure 2C) between EZL and HP-β-CD (affinity: −6.3 kcal/mol) demonstrated a hydrophobic interaction between the hydrogen on the benzene ring of EZL and the hydrogen on HP-β-CD. This indicates that the CD cavity is hydrophobic, making it easier for the benzene ring to enter. Generally, the interaction between EZL and the CDs was obtained through computer simulation: the C=O group on the EZL triazine ring forms intermolecular hydrogen bonds with the O-H group on the CDs, and there is a hydrophobic interaction between the hydrogen on EZL’s benzene ring and the hydrogen on the CDs. 

Furthermore, the data analysis revealed a linear association between the docking scores and *K*_f_ values. Similar results were reported by Matencio, A et al. [23]. This correlation highlights the simulated complex’s ability to capture fundamental subject–object interactions. Molecular simulations suggest that the high stability of the HP-β-CD complex stems from its greater hydrogen bonding and CD’s higher polarity. 

### 2.3. DSC Analysis

**Figure 3:**
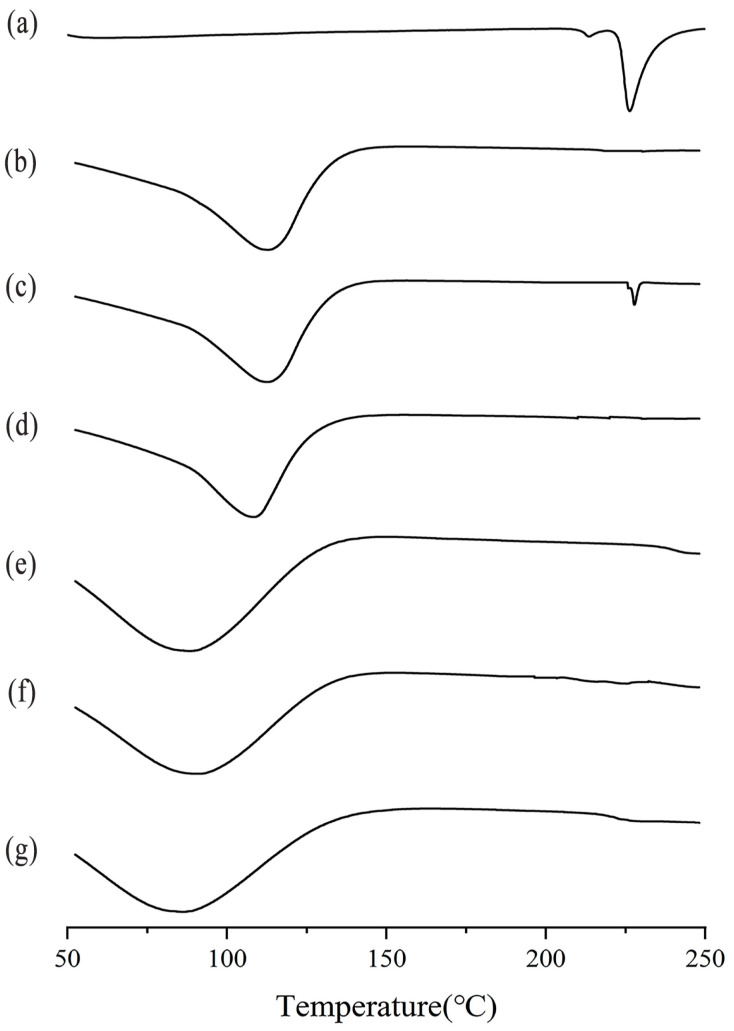
DSC thermograms of (**a**) EZL, (**b**) β-CD, (**c**) the EZL/β-CD physical mixture, (**d**) the EZL/β-CD inclusion complex, (**e**) HP-β-CD, (**f**) the EZL/HP-β-CD physical mixture, and (**g**) the EZL/HP-β-CD inclusion complex.

DSC research has shown to be a valuable method for investigating the solid-state interactions between drugs and CDs [24,25]. Figure 3 displays the DSC thermograms of EZL, β-CD, HP-β-CD, their physical mixtures, and the inclusion complexes. The DSC thermogram of pure EZL exhibited a heat absorption peak at 226 °C (Figure 3a), attributed to the melting point of EZL. 

The DSC thermograms of the physical mixtures of EZL/β-CD essentially showed a combination of the two components, indicating no complexation between EZL and β-CD (Figure 3c). The absence of the EZL melting peak in the thermogram of the EZL/β-CD inclusion complex, compared to the thermogram of the EZL/β-CD physical mixture, suggests the occurrence of inclusion [26]. This phenomenon may be due to the transformation of the crystalline form of EZL from crystalline to amorphous after the formation of inclusions [27,28].

Possibly due to the low EZL content and the sensitivity of the instrument, the characteristic melting peak of EZL is hardly distinguishable in the thermogram of the physical mixture of EZL/HP-β-CD (Figure 3f). Another possibility is that it produced a guest inclusion during the heating process. As a result, the formation of inclusion complexes of EZL/HP-β-CD cannot be determined through DSC detection.

### 2.4. PXRD Analysis

**Figure 4:**
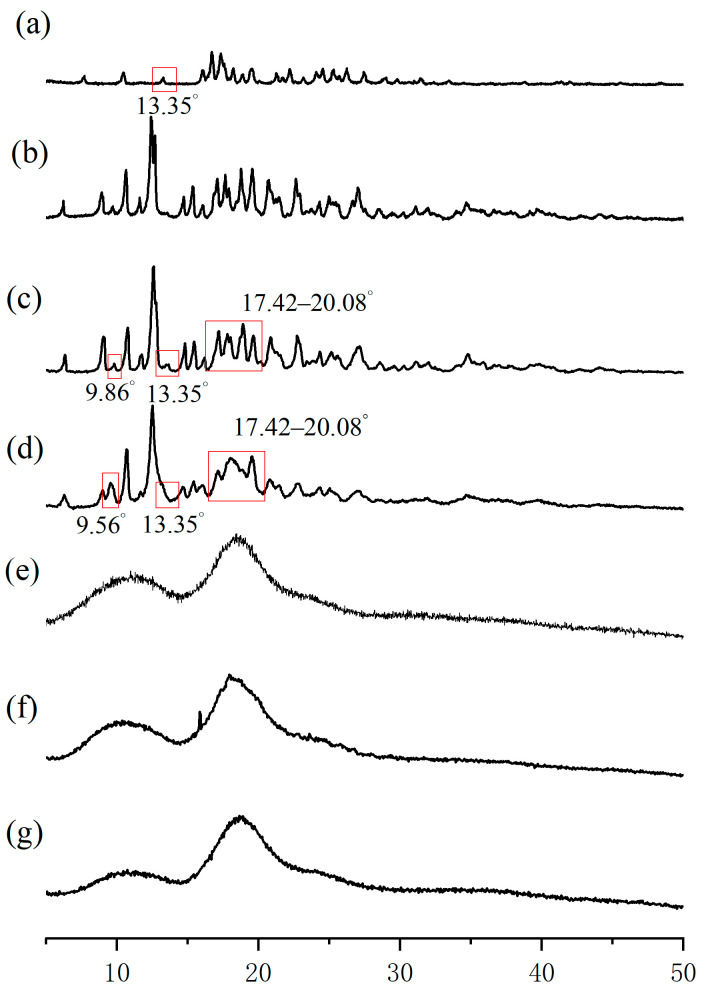
PXRD patterns of (**a**) EZL, (**b**) β-CD, (**c**) EZL/β-CD physical mixture, (**d**) EZL/β-CD inclusion complex, (**e**) HP-β-CD, (**f**) EZL/HP-β-CD physical mixture, and (**g**) EZL/HP-β-CD inclusion complex.

PXRD was employed to quantify the crystallinity of the complexes produced, and the location of the peaks serves as an indicator of the crystal structure [29,30]. Figure 4 displays the PXRD patterns of EZL, β-CD, HP-β-CD, their physical mixtures, and the inclusion complexes. EZL exhibited distinct sharp characteristic peaks at diffraction angles of 13.35°, 16.01°, 16.67°, 17.33°, and 18.18°. The β-CD molecule displayed its characteristic peaks at 8.90°, 10.59°, 12.61°, 17.72°, 18.81°, and 19.55°. The above information indicates that EZL and β-CD are crystalline states. HP-β-CD (Figure 4e) appeared amorphous, as there were no characteristic peaks in its PXRD pattern.

The PXRD patterns of the physical mixtures were investigated, and the peak positions matched those of the individual EZL and CDs (Figure 4g,f). This result indicates that there was no interaction between EZL and the respective CDs. Compared to the pattern of the EZL/β-CD physical mixture, in the pattern of the EZL/β-CD inclusion (Figure 4d), the characteristic diffraction peak of EZL (13.35°) disappeared and a new diffraction peak (9.56°) appeared [31]. Additionally, a broadening and weakening of the peak intensities were observed in the range of 17.42–20.08°, which strongly suggests the formation of the complex. Compared to the pattern of the EZL/HP-β-CD physical mixture, the pattern of the EZL/HP-β-CD inclusion (Figure 4g) showed a similar trend to amorphous HP-β-CD but did not display the characteristic features of EZL. This indicates that EZL was encapsulated by HP-β-CD. 

In addition, we can determine the solubility of the complexes based on their formation states. Solids with an amorphous structure have greater molecular mobility and apparent solubility compared to their crystalline counterparts [32]. Among the two CDs employed in this study, the EZL/HP-β-CD inclusion complex exhibited complete encapsulation, resulting in an amorphous appearance. Therefore, the EZL/HP-β-CD inclusion complex may have higher water solubility than the EZL/β-CD inclusion complex. This discovery corresponds with findings from phase solubility experiments.

### 2.5. FT-IR Spectra Analysis

The ATR technique can monitor even tiny changes in the status of the sample; unlike the KBr particle method, there is no sample preparation, and tiny changes in the status of the sample can be monitored when the milling and pressing predominantly give the sample weak interactions [33,34].

**Figure 5:**
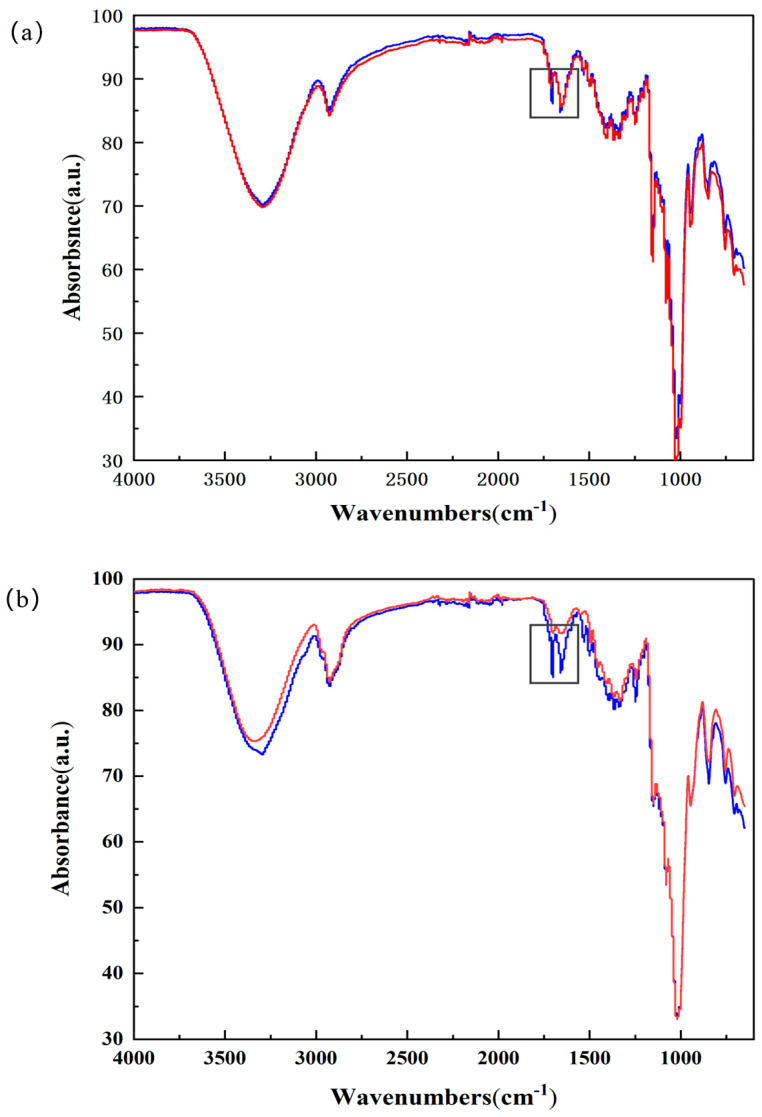
Overlap FT-IR spectra of (**a**) EZL/β-CD physical mixture and inclusion complex and (**b**) EZL/HP-β-CD physical mixture and inclusion complex (note: red–inclusion complex, blue–physical mixture).

FT-IR spectroscopy was utilized to evaluate the interaction between CDs and EZL in the solid state by examining the variations in the spectra generated by complexation [35]. We compared the FT-IR spectra of the EZL/CD physical mixture with those of the inclusion complex to determine the occurrence and mode of interaction between EZL and CDs. Comparatively, differences can be observed between the spectra. Interestingly, in the EZL/β-CD inclusion spectrum, the C=O absorption peak of EZL at 1700 cm^−1^ is not only weakened in intensity but also exhibits a slight blueshift to 1710 cm^−1^ when compared to the physical mixture spectrum (Figure 5a). Similarly, in the EZL/HP-β-CD inclusion spectrum, the C=O absorption peak at 1700 cm^−1^ was significantly weakened in intensity, without any observable shift (Figure 5b). The intermolecular hydrogen bond between the C=O group of EZL and the O-H group of the CDs is inferred to result in a change in the C=O absorption peak of EZL. This result is consistent with the results obtained by molecular docking.

### 2.6. UV–Vis Spectra Analysis

**Figure 6:**
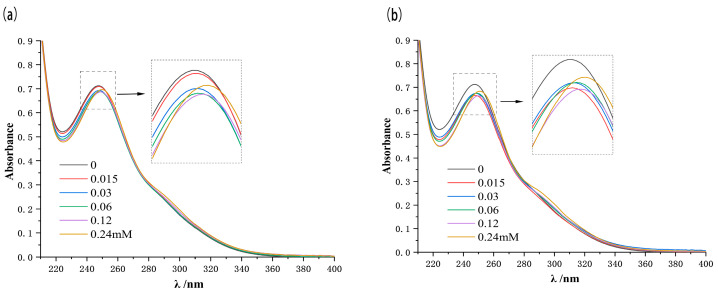
UV-vis spectral changes in EZL (0.03 mM) upon addition of β-CD (**a**: 0–0.24 mM) and HP-β-CD (**b**: 0–0.24 mM) in water at 25 °C.

The mutual interactions of EZL with the two CDs in solution were first investigated using UV-visible spectroscopy. In the spectrum of the EZL solution without CDs, the maximum absorption wavelength was located at 247 nm (Figure 6). As the concentration of CDs increased, the maximum absorption wavelength of EZL was slightly red-shifted from 247 nm to 251 nm. We tentatively hypothesize that this slight redshift may be due to the fact that EZL enters the CD cavity and is affected by the electron cloud in the CD cavity, which in turn leads to changes in the electron leaps of the conjugated groups on the structure of the EZL molecule.

### 2.7. MS Analysis

Since Ganem et al. first demonstrated that electrospray ionization mass spectrometry (ESI-MS) can be employed for the detection of noncovalent complexes [36,37], there has been a considerable amount of literature on the study of noncovalent CD complexes by ESI-MS [38,39,40,41]. 

**Figure 7:**
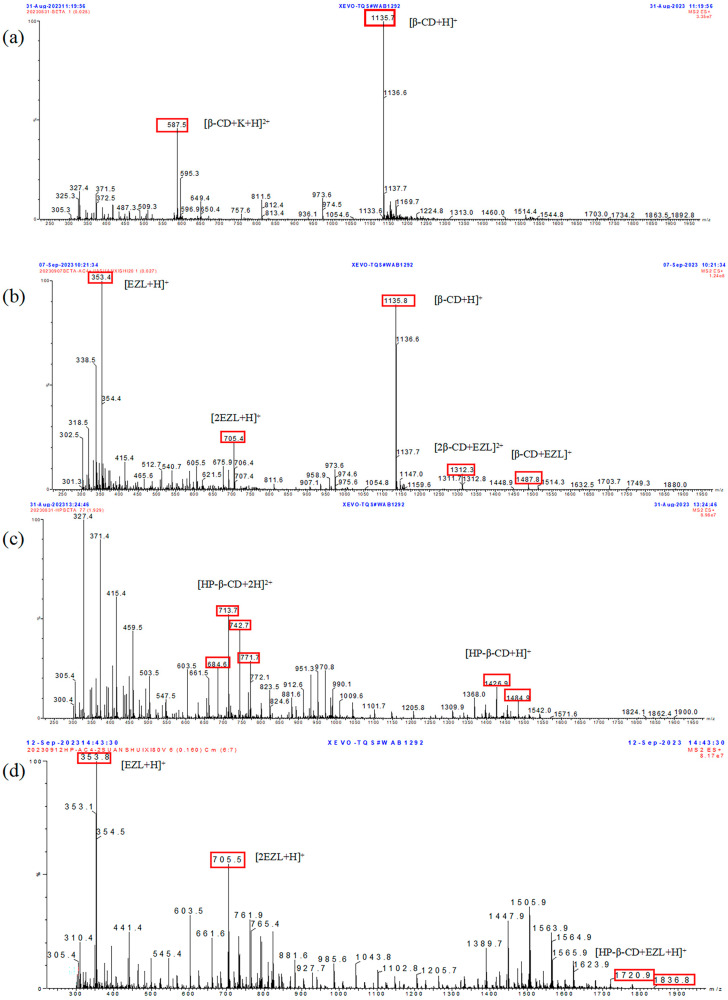
TQMS spectrum of (**a**) β-CD, (**b**) EZL/β-CD mixed solution, (**c**) HP-β-CD, and (**d**) EZL/HP-β-CD mixed solution.

In this study, we used TQMS equipped with an electrospray ionization source to assess inclusion formation and stoichiometry in the solution. Figure 4 exhibits the positive ESI mass spectrum of β-CD, HP-β-CD, EZL/β-CD mixed solution, and EZL/HP-β-CD mixed solution. In the spectrum of β-CD (Figure 7a), the peaks at *m*/*z* 587.5 and 1135.7 corresponded to [β-CD+K+H]^2+^ and [β-CD+H]^+^, respectively. For the EZL/β-CD mixed solution (Figure 7b), the peaks at *m*/*z* 353.4, 705.4, 1135.8, 1487.8, and 1312.8 corresponded to [EZL+H]^+^, [2EZL+H]^+^, [β-CD+H]^+^, [β-CD+EZL]^+^, and [2β-CD+EZL]^2+^, respectively. The above results demonstrate that both the 1:1 EZL/β-CD binding and the more complex 1:2 binding can be formed in solution.

As for HP-β-CD (Figure 7c), since HP-β-CD is a mixture with different amounts of hydroxypropyl groups, it exhibits a normal distribution of peaks with mass-to-charge ratio, and the peak with a double charge is located on the left side. The peaks at *m*/*z* 684.6, 713.7, 742.7, and 771.7 exhibited a 29 Da difference between adjacent peaks, indicating that the cluster corresponded to [HP-β-CD+2H]^2+^. The mass-to-charge peak of HP-β-CD on the right has a single charge. The peaks at *m*/*z* 1426.9 and 1484.9 exhibited a difference of 58 Da between adjacent peaks, suggesting that the cluster corresponded to [HP-β-CD+H]^+^. For the EZL/HP-β-CD mixed solution (Figure 7d), the peaks at *m*/*z* 1720.7 and 1836.8 exhibited a difference of 58 Da between adjacent peaks, indicating that the cluster corresponded to [HP-β-CD+EZL+H]^+^. These findings suggest the formation of a 1:1 EZL/HP-β-CD binding in solution.

In summary, the mass spectrometry results offer proof of the formation of distinct inclusion complexes in solution, including 1:1 and 2:1 binding for β-CD, as well as a 1:1 binding for HP-β-CD with EZL. This result is different from the 1:1 binding of EZL to both CDs, which we concluded from the phase solubility experiments described above. Our mass spectrometry study revealed the presence of a 2:1 binding between β-CD and EZL, in addition to the previously observed 1:1 binding. This may be attributed to a potential flaw in the mass spectrometry, which could result in false-positive outcomes. In ESI-MS experiments, extreme conditions (such as high electric fields and temperatures) can disrupt the chemical equilibrium of the system, resulting in aggregation and potentially influencing the determination of the true reaction state [42]. In other words, the binding of EZL and CDs in mass spectrometry may be attributed to both aggregation-induced reactions and gas-phase reactions during mass spectrometry, and there is no guarantee that the complexation occurred. Therefore, to further investigate the interaction between EZL and CDs, we conducted NMR studies.

### 2.8. ^1^H and 2D NMR Analysis

#### 2.8.1. ^1^H NMR Analysis

^1^NMR spectroscopy provides compelling evidence for the formation of inclusion complexes [43,44]. ^1^H NMR spectroscopic studies further validated the formation of the inclusion and identified potential interaction modes of the guest molecules in the inclusion structure [45,46].

**Figure 8:**
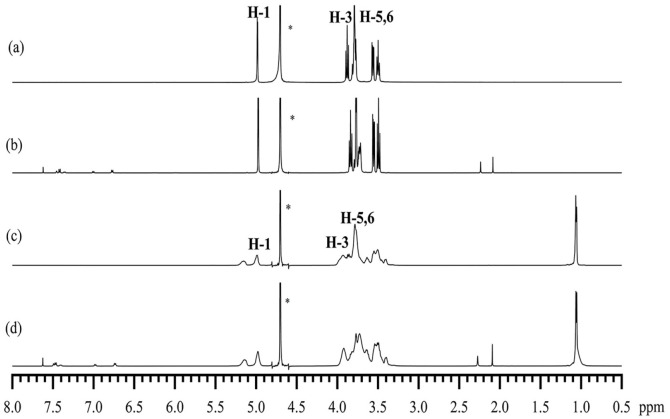
^1^H NMR spectra of (**a**) β-CD, (**b**) EZL/β-CD complex, (**c**) HP-β-CD, and (**d**) EZL/HP-β-CD complex (asterisk highlights the water peak).

In this study, we investigated the chemical shifts of the protons of β-CD and HP-β-CD in the presence and absence of EZL (Figure 8). After the formation of the EZL/β-CD and EZL/HP-β-CD complexes, the H-3 and H-5 protons located in the cavity exhibited upfield shifts, while the other CD protons (H-1, 2, 4, and 6) showed negligible changes in shift (Table 1). It is well known that if only the H-3 resonance is shifted in the presence of a substrate, it indicates a shallower penetration of the cavity. On the other hand, if the H-5 resonance is also shifted, it indicates a deeper penetration of the cavity [47]. These facts leave no doubt that EZL was deeply incorporated into the framework of the CDs. These upfield shifts are attributed to magnetic anisotropy effects in the CD cavities, which shield the protons in the CD cavities and cause them to show upfield shifts. The changes in the ^1^H NMR data clearly demonstrate the penetration of EZL molecules into the cavity of the CDs.

#### 2.8.2. 2D ROESY Analysis

2D ROESY is one of the most effective techniques for studying the intermolecular and intramolecular interactions of host–guests [45,48]. In ROESY, two protons within 0.4 nm of each other in space produce a nuclear Overhauser effect (NOE) cross-correlation, which gives substantial details on the structure of the inclusion complex [32,49]. 

**Figure 9:**
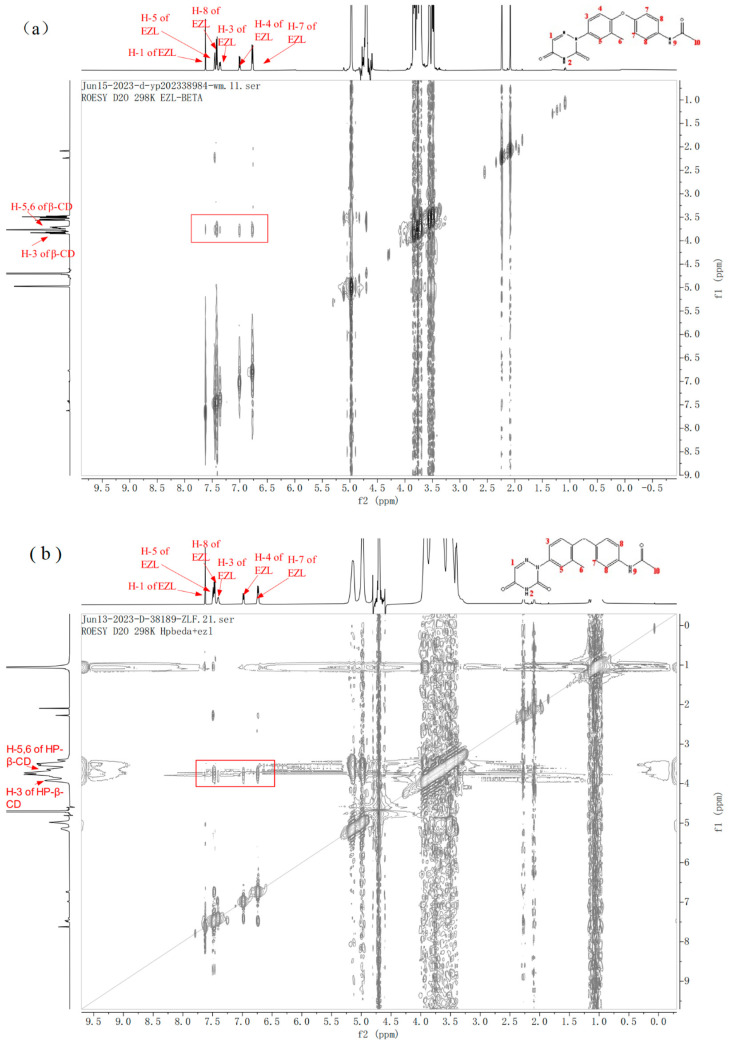
ROESY spectra of (**a**) EZL/β-CD complex and (**b**) EZL/HP-β-CD complex in D_2_O.

The 2D ROESY spectra for EZL/β-CD and EZL/HP-β-CD inclusion complexes are shown in Figure 9. The cross-peaks between the benzene ring protons of EZL (7.46–6.77 ppm) and the H-3 and H-5 protons in the β-CD and HP-β-CD cavities, as well as between H-1 on the EZL triazine ring and H-3 in the CDs cavities, are depicted in Figure 6. Thus, we conclude that EZL has penetrated the CD cavity, and the triazine ring of EZL and the benzene ring could potentially interact with CDs. This conclusion is also compatible with those derived from molecular docking.

## 3. Materials and Methods

### 3.1. Materials

Ethanamizuril (Lot No. 20230301) was prepared by the Animal Pharmacology Research Laboratory at the Shanghai Institute of Veterinary Medicine, Chinese Academy of Agricultural Sciences, Shanghai, China. β-CD (Lot No. 20230106, drying weight loss: 13.0%) and HP-β-CD (Lot No. 20230206, DS: 4.85, water content: 3.8%) were purchased from Shandong Binhai Zhiyuan Biotechnology Co (Shandong, China). The other chemicals and solvents utilized in this experiment were analytical or LC grade (China National Pharmaceutical Group Chemical Reagent Co., Shanghai, China). The solution was prepared and diluted using ultrapure water (18.2 MΩ·cm).

### 3.2. Phase Solubility Studies

First, we used the phase solubility method proposed by Higuchi and Connors to investigate the solubilizing effect of different *CDs* on *EZL* [20]. Excessive amounts of *EZL* were added to 10 mL of water containing varying concentrations of HP-β-CD (0–9 mM) or β-CD (0–9 mM). Suspensions were shaken at 25 °C for 48 h. After reaching equilibrium, all suspensions were filtered through a 0.22 μm membrane filter and analyzed using Waters e2695 high-performance liquid chromatography (Waters Corporation, Milford, CT, USA) at 250 nm. Phase solubility graphs were used to show how *EZL* solubility changed as *CD* concentration changed. We can obtain the formation constant (*K*_f_) and complexation efficiency (*CE*) between *EZL* and *CDs* from the phase solubility diagram. The stability constant *K*_f_ based on the slope of the linear region was calculated as follows:

(1) $$Kf=\frac{S\mathrm{lope}}{S_{0}(1-S\mathrm{lope})}$$

where *S*_0_ represents the intrinsic solubility of *EZL* without the presence of *CDs*. The solubilizing effect of *CD* could also be evaluated by computing the complexation efficiency (*CE*).

(2) $$CE=S_{0}\times K_{f}=\frac{\left[CD/EZL\right]}{\left[CD\right]}=\frac{S\mathrm{lope}}{1-S\mathrm{lope}}$$

### 3.3. Molecular Docking

In this study, we utilized the services provided by the Yinfo Cloud Platform (https://cloud.yinfotek.com/ (accessed on 3 October 2023)) to draw and optimize the 3D structures of the compounds, as well as conduct molecule docking. The 3D structure of EZL was drawn using free widgets provided by the Yinfo Cloud Platform. The 3D structure of β-CD was downloaded from the Cambridge Structural Database, with all associated water molecules removed. The initial structure of HP-β-CD was constructed by inserting seven hydroxypropyl moieties into the obtained β-CD. The EZL, β-CD, and HP-β-CD molecules were then energy optimized with the MMFF94 force field and saved in pdb format. The docking of molecules was carried out by applying Dock 6.9 and Vina., provided by the Yinfo Cloud Platform, with default parameters that considered flexible ligand docking. The optimal conformation of the inclusion compound was identified according to the configuration with the lowest binding energy in the system [50].

### 3.4. Preparation of the EZL/β-CD and EZL/HP-β-CD Systems in Solid State

Based on the previous PSA results, we determined the mass ratios of EZL to β-CD and HP-β-CD to be 1:21 and 1:18. Subsequently, the EZL/β-CD and EZL/HP-β-CD binary systems were obtained by using various methods as follows.

#### 3.4.1. Preparation of Inclusion Complexes by Shaking Bottle Method

The inclusion complexes were formed by dissolving EZL and CD in water with sufficient shaking in a constant temperature rocker rotator for at least 48 h at room temperature. Subsequently, the solutions were dried in a vacuum oven at 60 °C for 48 h.

#### 3.4.2. Preparation of Physical Mixtures

First, EZL and CD_S_ were separately ground into powder at room temperature. Then, the two compounds were lightly mixed in the proportions described above to achieve a homogeneous mixture.

### 3.5. DSC

On a DSC 2500 differential scanning calorimeter (TA Instruments, New Castle, DE, USA), we performed a DSC analysis. The reference for this test was an empty aluminum pan. The selected temperature range was 50~250 °C and the scan rate was 10 °C/min.

### 3.6. PXRD

Diffractograms of the solid inclusion complexes were obtained using a Bruker D8 ADVANCE Da Vinci multifunctional X-ray diffractometer (Bruker Corporation, Billerica, MA, USA) with Cu Kα radiation (40 kV, 15 mA) and 2θ scans at a scan rate of 8°/min. Diffraction data were collected over the angular range of 2θ = 2° to 50°. 

### 3.7. FT-IR

Solid samples were analyzed using FT-IR detection in attenuated total reflectance (ATR) geometry between 650 and 4000 cm^−1^. The spectra were obtained by applying a Nicolet iS10 FT-IR spectrometer (Thermo Scientific, Waltham, MA, USA). According to the ATR technology, the powders were put into the Golden Gate Diamond ATR system. The data were acquired in a dry environment, with a resolution of 4 cm^−1^ and an average of 16 scans.

### 3.8. UV–Vis Spectrophotometry

UV-vis spectra were obtained using an Agilent 8453 spectrophotometer (Agilent Technologies Inc., Santa Clara, CA, USA). The concentration of EZL was maintained constant at 0.03 mM. Adequate amounts of HP-β-CD or β-CD were then used with the final concentrations of 0, 0.015, 0.03, 0.06, 0.12, and 0.24 mM, respectively. UV-vis spectroscopy was then conducted in the spectral range of 200–400 nm.

### 3.9. MS

MS experiments were performed on a Waters Xevo TQ-S mass spectrometer (Waters Corporation, Milford, CT, USA). The samples of EZL/CD mixed solutions used in this analysis were obtained by dissolving the solid inclusion complexes prepared as described above in ultrapure water. The aqueous solutions were filtered and then tested. The MS conditions were as follows: electrospray ionization (ESI) in positive ion mode, desolvation temperature 200 °C, desolvation gas (N_2_) flow rate 400 L/h, capillary voltage setting 3.50 kV, and cone voltage setting 25 V. MS data were recorded in full scan mode, ranging from 300 to 1900 *m*/*z*. MassLynx 4.1 software was used to control the instruments and perform data acquisition and manipulation.

### 3.10. ^1^H NMR and 2D ROESY

A Bruker Avance AV-III 600 MHz spectrometer (Bruker Corporation, Billerica, MA, USA) was used to obtain ^1^H NMR and 2D ROESY spectra at room temperature. All samples were dissolved in D_2_O, respectively. The samples of EZL/CD complexes used in this analysis were obtained by dissolving the solid inclusion complexes prepared as described above in D_2_O. The aqueous solutions were filtered and then tested. This signal at 4.67 ppm of D_2_O served as an internal standard. Chemical shifts are represented in ppm.

## 4. Conclusions

In summary, the formation and mode of inclusion of the complexes were determined through detection in both liquid and solid states and molecular docking. The successful preparation of EZL/CD inclusion complexes was demonstrated by PSA, UV-vis spectroscopy, MS, DSC, and PXRD studies. The interaction between EZL and CDs was further investigated using NMR, FT-IR, and molecular docking. The results indicated that intermolecular hydrogen bonding between the C=O group on the triazine ring of EZL and the O-H group of CDs, as well as the hydrophobic interactions between the hydrogen on the benzene ring of EZL and the hydrogen of CDs, played crucial roles in the formation of EZL/CD inclusion complexes.

Through the preliminary pharmacodynamic study, it is known that the effective dose of EZL is 10 ug/mL [51]. According to the results of this study, both β-CD and HP-β-CD can effectively enhance EZL’s solubility. In the presence of 9 mM β-CD and HP-β-CD, the solubility of EZL was increased 37 and 58 times, respectively. Despite β-CD’s lower solubility, the affordability of β-CD makes it a practical choice for new product development. In conclusion, the results suggest that the two inclusions of EZL/CD could provide a promising technique for developing new dosage forms with high water solubility for future pharmacological applications.

## Figures and Tables

**Table 1:** The chemical shifts (δ) of the β-CD, HP-β-CD, EZL/β-CD, and EZL/HP-β-CD complexes.

		δ (ppm)			
		β-CD	EZL/β-CD Complex	HP-β-CD	EZL/HP-β-CD Complex
H-1	d	4.99	4.98	4.99	4.98
H-2	dd	3.57	3.56	3.55	3.54
H-3	dd	3.88	3.83	3.87	3.83
H-4	dd	3.50	3.49	3.50	3.49
H-5	m	3.77	3.72	3.72	3.64
H-6	dd	3.79	3.77	3.78	3.77
H-Me	s	-	-	1.07	1.05

## Data Availability

The data are contained within the article.
